# Production of *R-* and *S*-1,2-propanediol in engineered *Lactococcus lactis*

**DOI:** 10.1186/s13568-021-01276-8

**Published:** 2021-08-16

**Authors:** Rintaro Sato, Motoyuki Ikeda, Tomonari Tanaka, Hitomi Ohara, Yuji Aso

**Affiliations:** 1 Department of Biobased Materials Science, Kyoto Institute of Technology, Kyoto, Japan; 2 JST-Mirai Program, Japan Science and Technology Agency, Saitama, Japan

**Keywords:** Bioproduction, Fermentation, *Lactococcus lactis*, 1,2-Propanediol

## Abstract

1,2-propanediol (1,2-PDO) is a versatile chemical used in multiple manufacturing processes. To date, some engineered and non-engineered microbes, such as *Escherichia coli, Lactobacillus buchneri,* and *Clostridium thermosaccharolyticum,* have been used to produce 1,2-PDO. In this study, we demonstrated the production of *R*- and *S*-1,2-PDO using engineered *Lactococcus lactis*. The L- and D-lactic acid-producing *L. lactis* strains NZ9000 and AH1 were transformed with the plasmid pNZ8048-ppy harboring *pct*, *pduP*, and *yahK* genes for 1,2-PDO biosynthesis, resulting in *L. lactis* LL1 and LL2, respectively. These engineered *L. lactis* produced *S*- and *R*-1,2-PDO at concentrations of 0.69 and 0.50 g/L with 94.4 and 78.0% *ee* optical purities, respectively, from 1% glucose after 72 h of cultivation. Both 1% mannitol and 1% gluconate were added instead of glucose to the culture of *L. lactis* LL1 to supply NADH and NADPH to the 1,2-PDO production pathway, resulting in 75% enhancement of *S*-1,2-PDO production. Production of *S*-1,2-PDO from 5% mannitol and 5% gluconate was demonstrated using *L. lactis* LL1 with a pH–stat approach. This resulted in *S*-1,2-PDO production at a concentration of 1.88 g/L after 96 h of cultivation. To our knowledge, this is the first report on the production of *R*- and *S*-1,2-PDO using engineered lactic acid bacteria.

**Supplementary Information:**

The online version contains supplementary material available at 10.1186/s13568-021-01276-8.

## Introduction

Currently, sustainable manufacturing from biomass is in great demand to minimize negative environmental impacts (Abbasi and Abbasi 2010; Usmani et al. 2021). Bioproduction of a variety of industrial chemicals from biomass using microbes has been demonstrated (Tong et al. 1991; Huang et al. 2002, 2017; Nakamura and Whited 2003; Liang et al. 2011; Lin et al. 2016; Wang et al. 2017; Lee et al. 2018). For instance, Sato et al*.* demonstrated the direct production of 1,2-propanediol (1,2-PDO) and 1,3-propanediol from starch using engineered *Escherichia coli* BW25113 (Sato et al. 2020) and showed that inactivation of *glpF* improves 1,3-propanediol production (Sato et al. 2021).

Among diols, 1,2-PDO is one of the most versatile chemicals with two optical isomers, *R*-1,2-PDO and *S*-1,2-PDO (Altras and Cameron 1999; Niu et al. 2019). These compounds can be produced from glucose and/or xylose or lactic acid by engineered and non-engineered microbes. Some lactic acid bacteria (LAB) such as *Lactobacillus buchneri* and *Lactobacillus parabuchneri* produce 1,2-PDO (Elferink et al. 2001; Krooneman et al. 2002). *L. buchneri* produces 0.6 g/L of 1,2-PDO from 0.2% lactate (Elferink et al. 2001). Other researches demonstrated to produce the optical isomers of 1,2-PDO using microbes other than LAB (Cameron and Cooney 1986; Niu et al. 2019). For example, *Clostridium thermosaccharolyticum* HG-8 produced 3.8 and 3.2 g/L of *R*-1,2-PDO from 3% glucose and 3% xylose, respectively (Cameron and Cooney 1986). Engineered *E. coli* MG1655 possessing intrinsic or extrinsic lactate dehydrogenase (LDH) gene (*ldhA* or *lldh*), along with 1,2-PDO synthetic genes *pct*, *pduP*, and *yahK*, which encode propionate CoA-transferase, aldehyde dehydrogenase, and alcohol dehydrogenase, respectively, produced 17.3 g/L of *R*-1,2-PDO and 9.3 g/L of *S*-1,2-PDO from 4% glucose under fermenter-controlled cultivation conditions (Niu et al. 2019). 1,2-PDO can be produced in engineered microbes via a pathway in which glucose is first converted to D- and L-lactate, followed by the synthesis of *R*- and *S*-1,2-PDO from D- and L-lactate, respectively, in which cofactors such as acetyl-CoA, NADH, and NADPH are required (Altras and Cameron 1999; Saxena et al. 2010; Niu and Guo 2015; Niu et al. 2019). This suggests that microbes producing the 1,2-PDO precursor lactate at a high titer are suitable as production hosts for 1,2-PDO production, and that *R*- and *S*-1,2-PDO can be separately produced in D- and L-lactic acid producers, respectively. In fact, Niu et al. demonstrated the production of *R*- and *S*-1,2-PDO separately using D- and L-lactic acid-producing engineered *E. coli*, respectively (Niu et al. 2019).

Based on their glycolytic metabolism, LAB can be classified into two groups: homo- and hetero-fermentative LAB (Axelsson 2004; Bintsis 2018). Homofermentative LAB, such as *Lactococcus* spp., theoretically yield 2 mol of lactate from 1 mol of consumed glucose. LAB can also be divided into three groups: D-lactic acid producers, L-lactic acid producers, and both-isomer producers, which are determined by the catalytic properties of LDH encoded in their genomes (Gao et al. 2012; Rahman et al. 2013; Ghaffer et al. 2014; Eş et al. 2018). These results suggest that the use of homofermentative LAB as production hosts is reasonable for 1,2-PDO production, and that D- and L-lactic acid-producing LAB can be used to separate *R*- and *S*-1,2-PDO, respectively. Kuipers et al*.* constructed a recombinant host for the nisin-controlled gene expression (NICE) system, *L. lactis* NZ9000, by incorporating the two-component regulatory gene *nisRK* into the genome of *L. lactis* MG1363 (Kuipers et al. 1998). Since *L. lactis* NZ9000 has been used as a superior host for protein expression, this strain would be a suitable host for 1,2-PDO production. Recently, Aso et al*.* constructed a *L. lactis* NZ9000 derivative by replacing its major intrinsic L-LDH gene with a heterologous D-LDH gene from *Lactobacillus delbrueckii* subsp. *lactis* JCM 1107, resulting in a D-lactic acid producer, *L. lactis* AH1 (Aso et al. 2019). Using the AH1 strain, D-lactate was produced from starch directly by expression of a heterologous α-amylase gene from *Streptococcus bovis* NRIC 1535. From this demonstration, it is presumed that *R*- and *S*-1,2-PDO can be produced by the expression of *pct*, *pduP*, and *yahK* in *L. lactis* NZ9000 and AH1, respectively, using the NICE system.

**Fig. 1:**
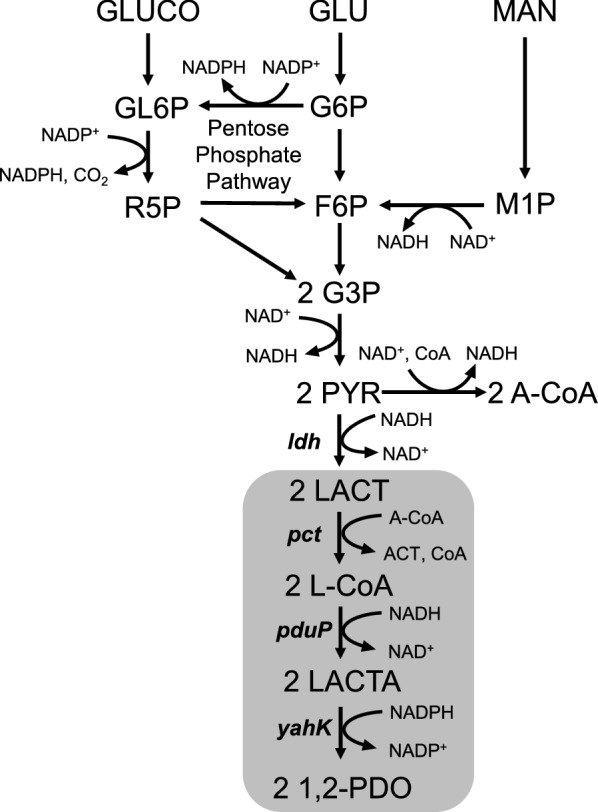
Metabolic pathway of 1,2-PDO production in engineered *L. lactis*. 1,2-PDO, 1,2-propanediol; G6P, glucose 6-phosphate; F6P, fructose-6-phosphate; G3P, glyceraldehyde-3-phosphate; M1P, mannitol-1-phosphate; GL6P, gluconate-6-phosphate; R5P, ribose-5-phosphate; PYR, pyruvate; A-CoA, acetyl-coenzyme A; ACT, acetate; GLU, glucose, GLUCO, gluconate; MAN, mannitol; LACT, lactate; L-CoA, lactoyl-CoA; LACTA, lactaldehyde. Italicized letters indicate genes: *ldh*, lactate dehydrogenase gene; *pct*, propionate CoA-transferase gene; *pduP*, aldehyde dehydrogenase gene; *yahK*, alcohol dehydrogenase gene. The introduced pathway for 1,2-PDO production is colored in gray

Therefore, the present study demonstrated the production of *R*- and *S*-1,2-PDO using engineered *L. lactis* NZ9000 and AH1, respectively, through an exogenous 1,2-PDO production pathway, according to a previously reported demonstration using engineered *E. coli* (Niu et al. 2019) (Fig. 1). Additionally, it has been reported that the metabolism of mannitol and gluconate in *L. lactis* results in enhancement of intracellular amounts of NADH and NADPH, respectively (Ramos et al. 2001; Neves et al. 2002; Wegmann et al. 2007; Linares et al. 2010). This suggests that addition of mannitol and gluconate improves 1,2-PDO production. To prove this, the effect of adding mannitol and gluconate to cultures of engineered *L. lactis* on 1,2-PDO production was investigated.

## Materials and methods

### Microorganisms and culture conditions

*Lactococcus lactis* NZ9000 (NIZO Food Research, Netherlands) (Kuipers et al. 1998) and AH1 (Aso et al. 2019) strains were grown in M17 medium (Difco Laboratories, MI, USA) supplemented with 0.5% glucose (GM17) at 30 °C. Chloramphenicol was added to the cultures at a final concentration of 20 µg/mL when required. For 1,2-PDO production, recombinant *L. lactis* strains were grown at 30 °C and 150 rpm under microaerobic conditions in M17 medium supplemented with 1% glucose, 1% gluconate, and/or 1% mannitol. When gluconate and mannitol were added to the medium, 0.3 g/L acetate was further added. In flask cultivation with 1% gluconate and 1% mannitol, an appropriate volume of 1 M MOPS (pH 7.0) was added to the culture every 24 h to adjust the pH of the culture to 7.0. Microaerobic conditions were performed in 200 mL flasks filled with 133 mL medium and sealed with rubber stoppers. Nisin A (Sigma-Aldrich, MO, USA) was added to the cultures at a final concentration of 10 ng/mL to induce gene expression for 1,2-PDO production when the optical density of cultures at 600 nm (OD_600_) reached 0.4–0.6.

To characterize production of L-lactate and *S*-1,2-PDO, resting cells of *L. lactis* LL1 were prepared as follows: recombinant *L. lactis* was cultivated in GM17 medium for 18 h at 30 °C. After centrifugation of the culture for 10 min, the culture supernatant was removed. Collected cells were resuspended in 1 mL of 100 mM sodium phosphate buffer (pH 7.0) containing 1% glucose, 1% sodium gluconate, and/or 1% mannitol after washing three times with distilled water. Resting cells were incubated for 72 h at 30 °C, and then the supernatant was subjected to analysis.

Fermentation controlled with a pH–stat was performed in a 2 L jar fermenter M-1000B (Tokyo Rikakikai Co., Ltd., Tokyo, Japan) with a working volume of 1 L at an agitation speed of 80 rpm at 30 °C without aeration under microaerobic conditions, and the pH of the culture was maintained at 6.8 by automatic addition of 2 M NaOH using a peristaltic pump. The concentration of dissolved oxygen (DO) in the culture was monitored using a field controller, model mk-750 DO (Automatic System Research Co., Ltd., Saytama, Japan).

### Genetic engineering

The plasmid pNZ8048-ppy for 1,2-PDO production was constructed as follows: a series of three genes, *pct* from *Megasphaera elsdenii* JCM 1772 (accession No. M26493), *pduP* from *Salmonella enterica* subsp. *enterica* NBRC 13245 (Accession No. AB680380), and *yahK* from *E. coli* BW25113 (Accession No. U00096), were amplified by PCR using the plasmid pSR5 (Sato et al. 2020) as a template with Q5 High-Fidelity DNA polymerase (New England Biolabs, MA, USA), and the primer set pct-F (5′-AATTATAAGGAGGCACTCACGAGGAGATATACCATGAGAAAAGTAGAAATCATTAC-3′) and yahK-R (5′-TACCGCATGCCTGCAGTACCTCAGTCTGTTAGTGTGCGATTATC-3′); the ribosome binding site (RBS) is underlined. The amplification reaction was as follows: denaturation at 98 °C for 2 min, 35 cycles of denaturation at 98 °C for 10 s, annealing at 60 °C for 30 s, and polymerization at 72 °C for 3 min. The pNZ8048 vector (Kuipers et al. 1998) was amplified by PCR using the primer set pNZ8048-F (5′-AAAGCAATTACTGATATTGCTGAAAAATTG-3′) and pNZ8048-R (5′-GGTGAGTGCCTCCTTATAATTTATTTTG-3′) under the same conditions. The amplified fragments were ligated by Gibson assembly (Gibson et al. 2009) with Gibson Assembly Master Mix (New England Biolabs), resulting in pNZ8048-ppy. The *pct*, *pduP*, and *yahK* genes were cloned downstream of each RBS (Additional file 1). *L. lactis* NZ9000 and AH1, which were transformed with pNZ8048-ppy by electroporation according to a previously described protocol (Guchte et al. 1992), resulting in *L. lactis* LL1 and LL2, respectively.

### Analytical methods

Concentrations of lactate, 1,2-PDO, glucose, gluconate, mannitol, acetate, and ethanol in the culture supernatants were measured using a Prominence HPLC system (Shimadzu, Kyoto, Japan) equipped with an Aminex HPX-87H (Bio-Rad, CA, USA) and a refractive index (RI) detector (Shimadzu). These analytes were eluted using a 5 mM sulfuric acid solution at a flow rate of 0.4 mL/min at 30 °C. The chirality of 1,2-PDO produced by *L. lactis* was determined using a Prominence HPLC system (Shimadzu) equipped with a CHIRAL ART Cellulose-C S-5 μm column (YMC Co., Ltd., Kyoto, Japan) and monitored using an RI detector (GL Science, Tokyo, Japan) according to a previously described protocol (Niu and Guo 2015). The mobile phase (99% hexane and 1% isopropanol) was eluted at a flow rate of 0.5 mL/min at 25 °C. The chirality of lactate produced by *L. lactis* was analyzed using a Prominence HPLC system (Shimadzu) equipped with an MCI GEL CRS10W column (Mitsubishi Chemical, Tokyo, Japan) and monitored at 254 nm using 1 mM copper (II) sulfate solution as the mobile phase at a 0.5 mL/min flow rate (Aso et al., 2019). The specific growth rate (*μ*) was calculated as the slope of the regression line, from a plot between ln (X/X_0_) and time (t) during the exponential growth period, where X (OD_600_) and X_0_ (OD_600_) are the cell concentrations at t (h) and at the beginning of the exponential phase, respectively.

## Results

### 1,2-PDO production in engineered *L. lactis*

Expression of proteins encoded by the *pct*, *pduP*, and *yahK* genes in engineered *L. lactis* was confirmed by SDS-PAGE analysis (data not shown). This showed that the NICE system induced expression of 1,2-PDO biosynthetic genes in engineered *L. lactis*. To check 1,2-PDO production in the engineered *L. lactis*, resting cells of *L. lactis* LL1 and LL2 were incubated for 72 h in a buffer containing 1% glucose (Table 1). After 72 h of incubation, *L. lactis* LL1 produced 0.62  ±  0.00 g/L of L-lactate and 0.69  ±  0.01 g/L of *S*-1,2-PDO, and *L. lactis* LL2 produced 0.52  ±  0.04 g/L of D-lactate and 0.73  ±  0.06 g/L of *R*-1,2-PDO. This showed that the introduced genes *pct*, *pduP*, and *yahK* function in both *L. lactis* NZ9000 and AH1. The optical purities of L-lactate and *S*-1,2-PDO produced by *L. lactis* LL1 were 96.8% and 94.4% *ee*, respectively, and the optical purities of D-lactate and *R*-1,2-PDO produced by *L. lactis* LL2 were 85.7% and 78.0% *ee*, respectively. This showed that *R*-and *S*-1,2-PDO can be separately produced in *L. lactis* LL1 and LL2, respectively.

**Table 1:** Production profiles of D- and L-lactate and *R*- and *S*-1,2-PDO in the engineered *L. lactis* after 72 h of incubation with 1% glucose

Strains	Production (g/L)				Optical purity (% *ee*)			
	D-lactate	L-lactate	*R*-1,2-PDO	*S*-1,2-PDO	D-lactate	L-lactate	*R*-1,2-PDO	*S*-1,2-PDO
*L. lactis* LL1	0.01 ± 0.00 (0.1 ± 0.01)^a^	0.62 ± 0.03 (6.15 ± 0.31)	0.02 ± 0.00 (0.20 ± 0.01)	0.69 ± 0.01 (6.90 ± 0.13)	–	96.8	–	94.4
*L. lactis* LL2	0.52 ± 0.03 (5.20 ± 0.01)	0.04 ± 0.01 (0.40 ± 0.05)	0.73 ± 0.04 (7.30 ± 0.04)	0.09 ± 0.01 (0.90 ± 0.14)	85.7	–	78.0	–

^a^Yield (%, g-product/g-glucose  ×  100)

**Fig. 2:**
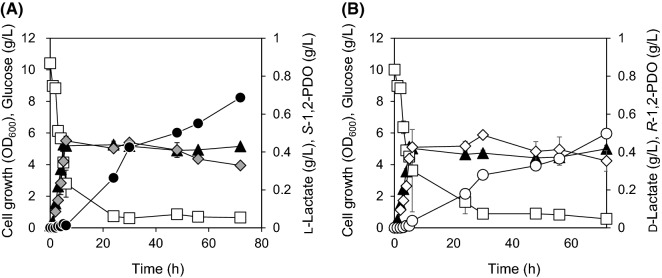
Production of 1,2-PDO in **A** *L. lactis* LL1 and **B** LL2 with 1% glucose in flask cultivation. The recombinants were cultivated in M17 medium supplemented with 1% glucose for 72 h under microaerobic conditions. Closed triangles, cell growth (OD_600_); closed circles, *S*-1,2-PDO; shaded diamonds, L-lactate; open circles, *R*-1,2-PDO; open diamonds, D-lactate; open squares, glucose. This experiment was performed in duplicate, and the average is represented with error bars

For characterization of 1,2-PDO production, *L. lactis* LL1 and LL2 were cultivated for 72 h in M17 medium supplemented with 1% glucose in a flask. After 72 h of cultivation, *L. lactis* LL1 produced 0.33  ±  0.00 g/L of L-lactate and 0.69  ±  0.01 g/L of *S*-1,2-PDO. *L. lactis* LL2 produced 0.35  ±  0.05 g/L of D-lactate and 0.50  ±  0.02 g/L of *R*-1,2-PDO (Fig. 2). The production profiles were similar to those obtained using resting cells. The 1,2-PDO yield from glucose in *L. lactis* LL1 was 1.4 times as high as in *L. lactis* LL2 (Table 2). The specific growth rates *μ* of *L. lactis* LL1 and LL2 were 1.02  ±  0.03 h^−1^ and 0.99  ±  0.01 h^−1^, respectively. There was no significant difference between OD_600_ at 72 h of cultivation and *μ* of *L. lactis* LL1 and LL2 (*P * >  0.05), indicating that the production of the optical isomers did not affect cell growth. Acetate and ethanol were detected as the main by-products in the cultures, but these production levels were negligible compared to those of 1,2-PDO (Table 2).

**Table 2:** Production profiles of metabolites in the engineered *L. lactis* after 72 h of cultivation with 1% glucose

Strains	OD_600_	Glucose consumption (g/L)	R-, S-1,2-PDO (g/L)	D-, L-lactate (g/L)	Acetate (g/L)	Ethanol (g/L)
*L. lactis* LL1	5.16 ± 0.06	9.36 ± 0.03	0.69 ± 0.00 (6.90 ± 0.01)^a^	0.33 ± 0.00 (3.30 ± 0.02)	0.23 ± 0.04 (2.46 ± 0.46)	0.04 ± 0.01 (0.42 ± 0.15)
*L. lactis* LL2	5.00 ± 0.22	9.43 ± 0.09	0.50 ± 0.02 (5.00 ± 0.01)	0.35 ± 0.05 (3.50 ± 0.03)	0.13 ± 0.01 (1.33 ± 0.13)	0.03 ± 0.02 (0.32 ± 0.15)

^a^Yield (%, g/g-glucose  ×  100)

### Supplementation of NADH and NADPH for 1,2-PDO production

**Fig. 3:**
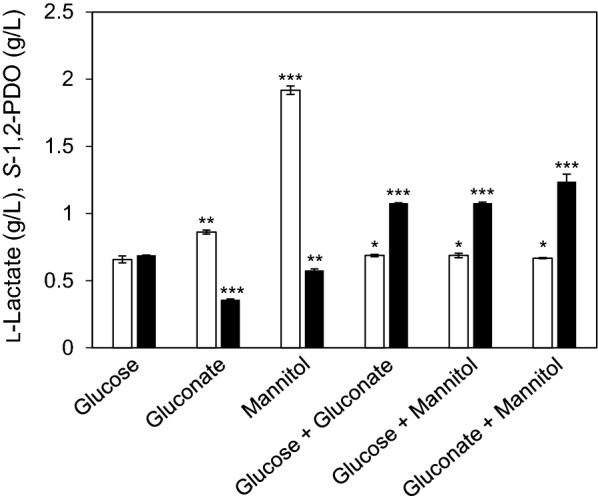
Characterization of *S*-1,2-PDO and L-lactate production in the resting cells of *L. lactis* LL1 incubated with 1% glucose, 1% mannitol, and/or 1% gluconate. The resting cells of *L. lactis* LL1 were incubated for 72 h at 30 °C in 100 mM phosphate buffer containing 1% glucose, 1% mannitol, and/or 1% gluconate. White bars, L-lactate; black bars, *S*-1,2-PDO. This experiment was performed in duplicate, and the average is represented with error bars. *P  >  0.05, **0.01  <  *P*  <  0.05, ***P  <  0.01 vs addition of only 1% glucose

To investigate the effect of adding gluconate and mannitol on 1,2-PDO production, resting cells of *L. lactis* LL1 were incubated in a buffer supplemented with 1% glucose, 1% mannitol, and/or 1% gluconate. The production levels of L-lactate and *S*-1,2-PDO from 1% glucose were 0.66  ±  0.01 g/L and 0.69  ±  0.01 g/L, respectively (Fig. 3). Interestingly, adding 1% gluconate significantly improved L-lactate production (0.80  ±  0.01 g/L), but *S*-1,2-PDO production was largely the same as when only glucose was added (0.58  ±  0.01 g/L). Addition of 1% mannitol alone led to the lowest *S*-1,2-PDO production (0.36  ±  0.01 g/L). On the other hand, addition of 1% mannitol or 1% gluconate with 1% glucose improved *S*-1,2-PDO production (1.08  ±  0.01 g/L and 1.08  ±  0.02 g/L, respectively). The highest production of *S*-1,2-PDO was obtained when both 1% mannitol and 1% gluconate without glucose were added (1.24  ±  0.01 g/L). These results indicate that NADH and NADPH were supplied through the metabolism of mannitol and gluconate, and consequently, 1,2-PDO production was improved.

**Fig. 4:**
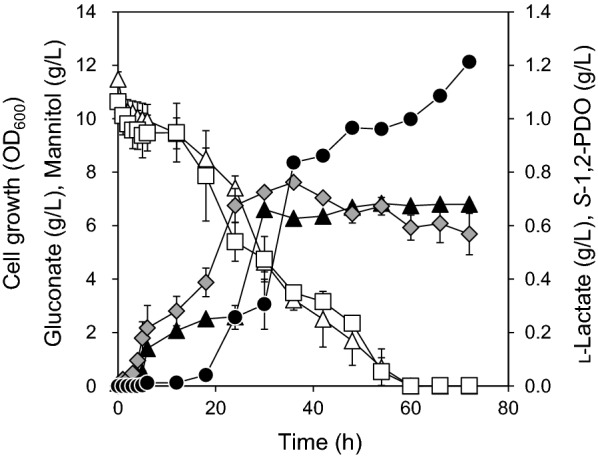
Production of 1,2-PDO in *L. lactis* LL1 with 1% gluconate and 1% mannitol in flask cultivation. *L. lactis* LL1 was cultivated for 72 h at 30 °C in M17 medium supplemented with 1% mannitol and 1% gluconate under microaerobic conditions. The medium was supplemented with 0.3 g/L acetate. The initial pH of the culture was 7.0. The culture was supplemented with 1 M MOPS (pH 7.0) every 24 h so that the pH of the culture was readjusted to 7.0. Closed triangles, cell growth (OD_600_); closed circles, *S*-1,2-PDO; shaded diamonds, L-lactate; open triangles, mannitol; open squares, gluconate. This experiment was performed in duplicate, and the average is represented with error bars

To characterize *S*-1,2-PDO production with gluconate and mannitol, *L. lactis* LL1 was cultivated with 1% mannitol and 1% gluconate for 72 h in a flask. The medium was supplemented with 0.3 g/L of acetate to enhance the intracellular level of acetyl-CoA in *L. lactis* (Puvendran and Jayaraman 2019). The initial pH of the culture was 7.0, but at 24 h of cultivation was 6.3. This was caused by L-lactate production, and resulted in 3.0 g/L of mannitol and 4.6 g/L of gluconate remaining in culture after cultivation. To promote consumption of mannitol and gluconate by *L. lactis* LL1, the pH of the culture was adjusted to 7.0 every 24 h by adding 1 M MOPS (pH 7.0). After 72 h of cultivation, *L. lactis* LL1 produced 0.57  ±  0.08 g/L of L-lactate and 1.21  ±  0.01 g/L of *S*-1,2-PDO from 1% mannitol and 1% gluconate (Fig. 4). The production titers of L-lactate and *S*-1,2-PDO were 1.7 times as high as those obtained with 1% glucose. The OD_600_ after 30 h of cultivation was 6.61  ±  0.06, and *μ* was 0.24  ±  0.00 h^−1^. These properties were comparable to those observed when cells were cultured with 1% glucose. This indicates that supplementation with mannitol and gluconate had similar effects on cell growth. Consumption rates of gluconate and mannitol were similar (gluconate, 0.19 g/L/h; mannitol, 0.18 g/L/h).

**Fig. 5:**
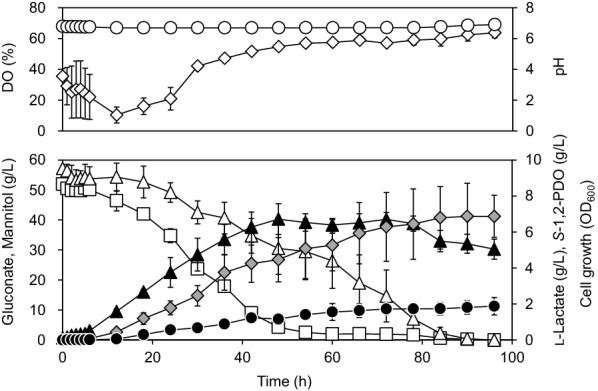
Production of 1,2-PDO in *L. lactis* LL1 from 5% mannitol and 5% gluconate in a jar fermenter controlled with a pH–stat. *L. lactis* LL1 was cultivated for 96 h at 30 °C in M17 medium supplemented with 5% mannitol and 5% gluconate under microaerobic conditions. The medium was supplemented with 0.3 g/L acetate. The pH of the culture was maintained at 6.8 by addition of 2 M NaOH during cultivation. Closed triangles, cell growth (OD_600_); closed circles, *S*-1,2-PDO; shaded diamonds, L-lactate; open triangles, mannitol; open squares, gluconate; open diamonds, concentration of DO in the culture; open circles, pH of the culture. This experiment was performed in duplicate, and the average is represented with error bars

It is known that maintaining the pH of *L. lactis* culture using a pH–stat method improves lactate production because a decrease in pH represses cell growth (Carvaiho et al. 2013). Therefore, we performed pH–stat-controlled production of *S*-1,2-PDO in M17 medium supplemented with 5% mannitol and 5% gluconate in a jar-fermenter. The medium was supplemented with 0.3 g/L acetate. Concentrations of the carbon sources were changed from 1 to 5% for prolonged cultivation because these compounds were depleted within 24 h of cultivation when 1% mannitol and 1% gluconate were used (data not shown). The pH of the culture was maintained near 6.8 during cultivation by the addition of 4 M NaOH (Fig. 5). The concentration of DO in the culture gradually decreased to 10.4% after 12 h of cultivation, and then remained almost constant after 48 h of cultivation (54.7–63.7%). *L. lactis* LL1 produced L-lactate (6.87  ±  1.13 g/L) and *S*-1,2-PDO (1.88  ±  0.44 g/L) after 96 h of cultivation. The OD_600_ after 48 h of cultivation was 6.72  ±  0.86, and *μ* was 0.15  ±  0.02 h^−1^. Compared to results obtained in flask cultivation, the production titers of L-lactate and *S*-1,2-PDO were 11.9 and 0.6 times higher, respectively. Gluconate was consumed first, and the consumption rate of gluconate was 1.8 times as high as that of mannitol during 54 h of cultivation.

## Discussion

Since the biosynthetic pathway for the production of 1,2-PDO has been reported, bioproduction of this compound has been attempted by introducing the biosynthetic pathway in various microorganisms including *E. coli* (Cameron and Cooney 1986, Niu and Guo 2015, Niu et al. 2019). In this study, we demonstrated production of *R*- and *S*-1,2-PDO using engineered *L. lactis* constructed from L- and D-lactic acid-producing LAB, *L. lactis* NZ9000, and AH1, respectively, by expression of 1,2-PDO biosynthetic genes with glucose, mannitol, and/or gluconate (Fig. 1). The advantage of using LAB is that lactic acid is the main organic acid required for LAB to produce the desired product, 1,2-PDO.

The resting cells of *L. lactis* LL1 produced L-lactate and *S*-1,2-PDO at concentrations of 0.62 g/L and 0.69 g/L, respectively, with negligible production of other isomers (Table 1). On the other hand, *L. lactis* LL2 produced an isomer each of lactate and 1,2-PDO (Table 1). This is because *L. lactis* AH1, which is the parent strain of *L. lactis* LL2, has been reported to produce D-lactate at an optical purity of 87.5% *ee* (Aso et al. 2019). We obtained a similar result, in that the optical purity of D-lactate was 85.7% *ee* when *L. lactis* LL2 was cultivated in this study. These results indicate that the optical purities of *R*- and *S*-1,2-PDO produced by the engineered *L. lactis* were determined in accordance with those of the D- and L-lactate produced. The low D-lactate optical purity of *L. lactis* LL2 is due to the fact that *L. lactis* AH1 has *lldh* homologue genes (*ldh*B, *ldh*X, and *hic*D) in addition to *lldh* (Aso et al. 2019). The production of lactate, a primary metabolite, is known to be associated with cell growth. Actually, however, the resting cells of *L. lactis* LL1 and LL2 produced lactate and 1,2-PDO from glucose. This is may be due to that *L. lactis* MG1363, which is the parent strain of *L. lactis* LL1 and LL2, maintains enzymatic activity related to the glycolysis during all phases of cell growth (Palmfeldt et al. 2004). ATP was probably pooled in the resting cells. Because, it has been reported that ATP in the resting cells of *L. lactis* was increased compared to the growing cells (Palmfeldt et al. 2004).

Production of *R*- and *S*-1,2-PDO in the growing cells of *L. lactis* LL1 and LL2 with 1% glucose at 72 h of cultivation was characterized. Production of 1,2-PDO by *L. lactis* LL1 was 1.4 times as high as that produced by *L. lactis* LL2 (Fig. 2). Interestingly, production of 1,2-PDO by *L. lactis* LL1 was more than 40 times as high as that produced by a *pflA*-deficient *E. coli* BW25113, harboring the same set of 1,2-PDO biosynthetic genes, from 1% glucose, as previously reported (Sato et al. 2020). The engineered *E. coli* produced heterofermentatively D-lactate along with acetate and ethanol at non-negligible concentrations, leading to the decrease of the yield of D-lactate. On the other hand, engineered *E. coli* that produced the maximum 1,2-PDO titer, showed 1.05 g/L acetate, and 0.13 g/L ethanol production from 1% glucose (Niu et al. 2019). In contrast, homofermentative LAB, including *L. lactis*, produce lactate with by-products at negligible concentrations. Therefore, 1,2-PDO production by engineered *L. lactis* significantly increased compared to that produced by the engineered the engineered *pflA*-deficient *E. coli* BW25113. *L. lactis* effectively produces lactate from glucose under microaerobic conditions (Papagianni et al. 2007). *L. lactis* is cultured under aerobic conditions, the lactate production is low due to NADH oxidase. This results in the production of acetate (Neves et al. 2002). On the other hand, 1 mol each of acetyl-CoA, NADH, and NADPH are required for 1 mol of 1,2-PDO production. To supply acetyl-CoA, microaerobic conditions were demonstrated in this study. Although this study demonstrated to produce 1,2-PDO under microaerobic condition, the lactate titer of *L. lactis* LL1 and LL2 was lower than that of *L. lactis* NZ9000 and AH1 which produced 7.0 g/L and 6.6 g/L of lactate from 1% glucose, respectively (Aso et al. 2019). This result indicated that the overexpression of *pct*, *pduP*, and *yahK* induced the conversion of lactate to 1,2-PDO. Overexpression of phosphofructokinasegene (*pfkA*) from *Aspergillus niger* in *L. lactis* LM0230 improved glucose uptake and enhanced lactate production yields (Papagianni and Avramidis 2011). Therefore, the overexpression of *pfkA* gene in *L. lactis* LL1 and LL2 might be improved lactate and 1,2-PDO productions.

It is known that additional NADH and NADPH are generated in *L. lactis* through the metabolism of gluconate and mannitol, and that mannitol-1-phosphate dehydrogenase and 6-phospogluconate dehydrogenase generate NADH and NADPH, respectively (Ramos et al. 2001; Neves et al. 2002; Wegmann et al. 2007; Linares et al. 2010; Zhao et al., 2017). To improve 1,2-PDO production by supplying these cofactors to the 1,2-PDO biosynthetic pathway, effects of adding glucose, mannitol, and/or gluconate on 1,2-PDO and lactate production using the resting cells of *L. lactis* LL1 were investigated. Although addition of either 1% mannitol or gluconate led to decreased *S*-1,2-PDO production compared to addition of 1% glucose, addition of both compounds resulted in the highest *S*-1,2-PDO production (Fig. 3). Mannitol is intracellularly converted to mannitol-1-phosphate, followed by the formation of fructose-6-phosphate. Fructose-6-phosphate is not metabolized via the pentose phosphate pathway, in which NADPH is generated. Therefore, the addition of 1% mannitol alone led to the lowest *S*-1,2-PDO production. It was suggested that the addition of gluconate alone does not contribute to the cellular redox balance and consequently results in a decrease in *S*-1,2-PDO production. Interestingly, the production of L-lactate was significantly enhanced by the addition of 1% gluconate. This may be because sodium ions derived from gluconate neutralize the L-lactate produced, and then the resting cells produce a large amount of L-lactate (1.92 g/L).

Production of *S*-1,2-PDO was characterized by cultivation of *L. lactis* LL1 with M17 medium supplemented with 1% gluconate and 1% mannitol in flask cultivation. The use of 1% gluconate and 1% mannitol led to 75% enhancement of *S*-1,2-PDO production than did the use of 1% glucose (Fig. 4). In the metabolic pathway in the engineered *L. lactis*, 1 mol of NADH are synthesized from 1 mol of mannitol whereas 1 mol of NADH and 1 mol of NADPH are synthesized from 1 mol of gluconate (Niu et al. 2019) (Fig. 1). In the following step, the synthetic pathway for 1,2-PDO production consumes the equivalent numbers of NADH and NADPH. This ensures the redox balance for the whole process. This proves that 1,2-PDO production is enhanced by an additional supply of NADH and NADPH derived from mannitol and gluconate in the engineered *L. lactis*. Niu and Guo have also mentioned the necessity of supplying NADH and NADPH for 1,2-PDO production in recombinant *E. coli* (Niu and Guo 2015; Niu et al. 2019).

Generally, lactate production by LAB can be improved by maintaining the pH of the cultures during fermentation (Andersen et al., 2009; Carvaiho et al. 2013). Therefore, jar fermenter cultivation with a pH–stat for *S*-1,2-PDO production was performed with 5% gluconate and 5% mannitol. As a result, the production of lactate was improved, but the production of 1,2-PDO did not significantly increase (Fig. 5). In flask cultivation with 1% mannitol and 1% gluconate, there is no significant difference between the consumption rates of gluconate and mannitol (gluconate, 0.19 g/L/h; mannitol, 0.18 g/L/h). However, the consumption rate of gluconate was 1.8 times as high as that of mannitol in the jar-fermenter cultivation with 5% mannitol and 5% gluconate during 54 h of cultivation (gluconate, 0.91 g/L/h; mannitol, 0.51 g/L/h). Neves et al. reported that the NADH burden caused by mannitol-1-phosphate dehydrogenase inhibits mannitol metabolism in *L. lactis* MG1363 cultivated with mannitol (Neves et al. 2002). On the other hand, gluconate seems to be metabolized through the pentose phosphate pathway and glycolysis without such obstruction because NADPH generated from gluconate is smoothly consumed for the synthesis of cell biomass, especially during the exponential growth phase. This suggests that gluconate is more easily metabolized than mannitol when added at a higher concentration (5%), and consequently, gluconate is consumed first. The production rate of *S*-1,2-PDO was nearly constant after 48 h of cultivation. This may be because gluconate in the culture was almost completely consumed after 48 h of cultivation. Instead of the addition of gluconate, overexpression of fructose 1,6-bisphosphatase gene (*fbp*) and glucose-6-phosphate dehydrogenase gene (*zwf*) for NADPH supply might be possible.

## Supplementary Information

**Additional file 1: **The partial sequence of pNZ8048-ppy.

## Data Availability

The authors can confirm that all relevant data are included in the article.
