# Supplementary material for: Production of R- and S-1,2-propanediol in engineered Lactococcus lactis
Source: AMB Express. 2021 Aug 16;11:117. doi: 10.1186/s13568-021-01276-8 (PMC8368392; doi:10.1186/s13568-021-01276-8)
Supplement: Supplementary file 1 — Additional file 1: The partial sequence of pNZ8048-ppy. [file 13568_2021_1276_MOESM1_ESM.docx]

**Supplementary Information**

Applied Microbiology and Biotechnology Express

Production of *R-* and *S*-1,2-propanediol in engineered *Lactococcus lactis*

Rintaro Sato^1,2^, Motoyuki Ikeda^1^, Tomonari Tanaka^1^, Hitomi Ohara^1^, Yuji Aso^1,2^

^1^ Department of Biobased Materials Science, Kyoto Institute of Technology, Kyoto, Japan

^2^ JST-Mirai Program, Japan Science and Technology Agency, Saitama, Japan

Corresponding author: Yuji Aso

E-mail: aso@kit.ac.jp

**The partial sequence of pNZ8048-ppy**

The *nisA* promoter is highlighted in yellow. The ribosome binding site is shown in bold. The primer sequences used for the construction of the plasmid are underlined. Red, *pct* gene; blue, *pduP* gene; green, *yahK* gene.

GGTAATAATATTATTGTCGATAACGCGAGCATAATAAACGGCTCTGATTAAATTCTGAAGTTTGTTAGATACAATGATTTCGTTCGAAGGAACTACAAAATAAATTATAAGGAGGCACTCACG**AGGAGA**TATACCATGAGAAAAGTAGAAATCATTACAGCTGAACAAGCAGCTCAGCTCGTAAAAGACAACGACACGATTACGTCTATCGGCTTTGTCAGCAGCGCCCATCCGGAAGCACTGACCAAAGCTTTGGAAAAACGGTTCCTGGACACGAACACCCCGCAGAACTTGACCTACATCTATGCAGGTTCTCAGGGCAAACGCGACGGCCGTGCTGCTGAACATCTGGCACATACAGGTCTTTTGAAACGTGCCATCATCGGCCACTGGCAGACAGTACCGGCTATCGGTAAACTGGCTGTCGAAAACAAAATCGAAGCTTACAACTTTTCGCAGGGTACGTTGGTCCACTGGTTCCGCGCCTTGGCAGGTCACAAGCTCGGCGTCTTCACGGACATCGGCCTGGAAACCTTCTTGGATCCTCGTCAACTCGGCGGTAAGCTCAATGACGTAACGAAAGAAGACTTGGTCAAATTGATTGAAGTCGATGGTCATGAACAGCTTTTCTATCCGACCTTTCCGGTCAACGTAGCTTTCCTCCGCGGCACCTATGCCGATGAATCCGGCAACATCACGATGGACGAAGAAATCGGGCCTTTTGAAAGCACTTCCGTAGCCCAGGCCGTTCACAACTGTGGCGGTAAAGTCGTCGTCCAGGTCAAAGATGTCGTTGCTCACGGCAGCCTCGACCCGCGCATGGTCAAGATTCCTGGCATCTATGTTGATTACGTTGTCGTAGCTGCTCCGGAAGACCATCAGCAGACGTATGACTGCGAATATGATCCGTCCCTTAGCGGCGAACATCGTGCTCCTGAAGGCGCTGCTGACGCAGCTCTCCCGATGAGCGCTAAGAAAATCATCGGTCGCCGCGGTGCTTTGGAATTGGCCGAAAACGCTGTCGTCAACCTCGGCGTCGGCGCTCCGGAATACGTTGCTTCCGTTGCTGGTGAAGAAGGTATCGCTGATACCATTACCTTGACCGTCGAAGGTGGCGCTATCGGTGGTGTACCGCAGGGCGGTGCCCGCTTCGGTTCGTCCCGTAATGCTGATGCCATCATCGACCATACTTATCAGTTCGACTTCTACGATGGCGGCGGCTTGGACATTGCTTACCTCGGCTTGGCTCAGTGCGATGGTTCGGGCAACATCAACGTCAGCAAGTTCGGTACGAACGTTGCCGGTTGCGGCGGTTTCCCGAATATCTCCCAGCAGACACCGAATGTTTACTTCTGCGGCACGTTCACAGCTGGCGGCTTGAAAATCGCTGTTGAAGATGGCAAAGTCAAAATCCTCCAGGAAGGCAAAGCCAAGAAGTTCATCAAAGCAGTCGATCAGATTACTTTCAACGGTTCTTATGCAGCCCGCAACGGCAAACACGTCCTCTACATCACGGAACGCTGCGTATTCGAACTGACTAAAGAAGGCTTGAAACTCATCGAAGTCGCACCGGGCATTGATATTGAAAAAGATATCCTCGCTCACATGGACTTCAAGCCGATTATTGATAATCCGAAACTCATGGATGCCCGCCTCTTCCAGGACGGTCCCATGGGACTGAAAAAATATATAG**AGGAGA**TATACCATGAATACTTCTGAACTCGAAACCCTGATTCGCACCATTCTTAGCGAGCAATTAACCACGCCGGCGCAAACGCCGGTCCAGCCTCAGGGCAAAGGGATTTTCCAGTCCGTGAGCGAGGCCATCGACGCCGCGCACCAGGCGTTCTTACGTTATCAGCAGTGCCCGCTAAAAACCCGCAGCGCCATTATCAGCGCGATGCGTCAGGAGCTGACGCCGCTGCTGGCGCCCCTGGCGGAAGAGAGCGCCAATGAAACGGGGATGGGCAACAAAGAAGATAAATTTCTCAAAAACAAGGCTGCGCTGGACAACACGCCGGGCGTAGAAGATCTCACCACCACCGCGCTGACCGGCGACGGCGGCATGGTGCTGTTTGAATACTCACCGTTTGGCGTTATCGGTTCGGTCGCCCCAAGCACCAACCCGACGGAAACCATCATCAACAACAGTATCAGCATGCTGGCGGCGGGCAACAGTATCTACTTTAGCCCGCATCCGGGAGCGAAAAAGGTCTCTCTGAAGCTGATTAGCCTGATTGAAGAGATTGCCTTCCGCTGCTGCGGCATCCGCAATCTGGTGGTGACCGTGGCGGAACCCACCTTCGAAGCGACCCAGCAGATGATGGCCCACCCGCGAATCGCAGTACTGGCCATTACCGGCGGCCCGGGCATTGTGGCAATGGGCATGAAGAGCGGTAAGAAGGTGATTGGCGCTGGCGCGGGTAACCCGCCCTGCATCGTTGATGAAACGGCGGACCTGGTGAAAGCGGCGGAAGATATCATCAACGGCGCGTCATTCGATTACAACCTGCCCTGCATTGCCGAGAAGAGCCTGATCGTAGTGGAGAGTGTCGCCGAACGTCTGGTGCAGCAAATGCAAACCTTCGGCGCGCTGCTGTTAAGCCCTGCCGATACCGACAAACTCCGCGCCGTCTGCCTGCCTGAAGGCCAGGCGAATAAAAAACTGGTCGGCAAGAGCCCATCGGCCATGCTGGAAGCCGCCGGGATCGCTGTCCCTGCAAAAGCGCCGCGTCTGCTGATTGCGCTGGTTAACGCTGACGATCCGTGGGTCACCAGCGAACAGTTGATGCCGATGCTGCCAGTGGTAAAAGTCAGCGATTTCGATAGCGCGCTGGCGCTGGCCCTGAAGGTTGAAGAGGGGCTGCATCATACCGCCATTATGCACTCGCAGAACGTGTCACGCCTGAACCTCGCGGCCCGCACGCTGCAAACCTCGATATTCGTCAAAAACGGCCCCTCTTATGCCGGGATCGGCGTCGGCGGCGAAGGCTTTACCACCTTCACTATCGCCACACCAACCGGTGAAGGGACCACGTCAGCGCGTACTTTTGCCCGTTCCCGGCGCTGCGTACTGACCAACGGCTTTTCTATTCGCTAA**AGGAGA**TATACCATGAAGATCAAAGCTGTTGGTGCATATTCCGCTAAACAACCACTTGAACCGATGGATATCACCCGGCGTGAACCGGGACCGAATGATGTCAAAATCGAAATCGCTTACTGTGGCGTTTGCCATTCCGATCTCCACCAGGTCCGTTCCGAGTGGGCGGGGACGGTTTACCCCTGCGTGCCGGGTCATGAAATTGTGGGGCGTGTGGTAGCCGTTGGTGATCAGGTAGAAAAATATGCGCCGGGCGATCTGGTCGGTGTCGGCTGCATTGTCGACAGTTGTAAACATTGCGAAGAGTGTGAAGACGGGTTGGAAAACTACTGTGATCACATGACCGGCACCTATAACTCGCCGACGCCGGACGAACCGGGCCATACTCTGGGCGGCTACTCACAACAGATCGTCGTTCATGAGCGATATGTTCTGCGTATTCGTCACCCGCAAGAGCAGCTGGCGGCGGTGGCTCCTTTGTTGTGTGCAGGGATCACCACGTATTCGCCGCTACGTCACTGGCAGGCCGGGCCGGGTAAAAAAGTGGGCGTGGTCGGCATCGGCGGTCTGGGACATATGGGGATTAAGCTGGCCCACGCGATGGGGGCACATGTGGTGGCATTTACCACTTCTGAGGCAAAACGCGAAGCGGCAAAAGCCCTGGGGGCCGATGAAGTTGTTAACTCACGCAATGCCGATGAGATGGCGGCTCATCTGAAGAGTTTCGATTTCATTTTGAATACAGTAGCTGCGCCACATAATCTCGACGATTTTACCACCTTGCTGAAGCGTGATGGCACCATGACGCTGGTTGGTGCGCCTGCGACACCGCATAAATCGCCGGAAGTTTTCAACCTGATCATGAAACGCCGTGCGATAGCCGGTTCTATGATTGGCGGCATTCCAGAAACTCAGGAGATGCTCGATTTTTGCGCCGAACATGGCATCGTGGCTGATATAGAGATGATTCGGGCCGATCAAATTAATGAAGCCTATGAGCGAATGCTGCGCGGTGATGTGAAATATCGTTTTGTTATCGATAATCGCACACTAACAGACTGAAAAGCAATTACTGATATTGCTGAAAAATTG
